# Editorial: Role of the SUMOylation in cancer regulation

**DOI:** 10.3389/fmolb.2023.1236230

**Published:** 2023-08-31

**Authors:** Xu Chen, Giovanni Smaldone, Pier Paolo Piccaluga, Yitao Qi

**Affiliations:** ^1^ Key Laboratory of the Ministry of Education for Medicinal Resources and Natural Pharmaceutical Chemistry, College of Life Sciences, Shaanxi Normal University, Xi’an, Shaanxi, China; ^2^ National Engineering Laboratory for Resource Developing of Endangered Chinese Crude Drugs in Northwest China, College of Life Sciences, Shaanxi Normal University, Xi’an, Shaanxi, China; ^3^ IRCCS SYNLAB SDN, Naples, Italy; ^4^ Department of Experimental, Diagnostic, and Specialty Medicine, Bologna University School of Medicine, Bologna, Italy

**Keywords:** SUMOylation, cancer regulation, biomarker, potential diagnosis and prognosis, treatment

In this Research Topic “*Role of the SUMOylation in Cancer Regulation*” there is a collection of original comprehensive research and reviews that describe the role of SUMOylation in cancer regulation. SUMOylation is a post-translational modification process that involves the covalent conjugation of SUMO proteins to specific target proteins (Chang and Yeh, 2020). This process is critical in regulating various essential cellular processes, such as gene expression (Rodriguez-Castaneda et al., 2018; Ryu and Hochstrasser, 2021), DNA damage repair (Bergink and Jentsch, 2009; Ryu and Hochstrasser, 2021), and protein homeostasis (Da Silva-Ferrada et al., 2016). Dysregulation of SUMOylation has been linked to the pathogenesis and progression of various types of cancer, underscoring its significance in cancer biology (Chang and Yeh, 2020; Chen et al., 2021; Qin et al., 2021). Therefore, the SUMOylation pathway has gained increasing attention as a potential regulatory switch and a promising therapeutic target for cancer treatment. Given the critical regulatory role of SUMOylation in cancer biology and the latest research development in the field, we organized this Research Topic to provide a comprehensive summary of the new mechanisms of SUMOylation in the regulation of tumor development. The following is the summary of the original research and reviews from each contributor for this Research Topic of “*Role of the SUMOylation in Cancer Regulation*”.

In the first article in this Research Topic, Li et al. identified UBE2I as a potential novel biomarker for clear cell renal cell carcinoma (ccRCC) using a large-scale CRISPR-Cas9 screening database and immunohistochemistry (Li et al.). This study provides new insights into the molecular mechanisms underlying ccRCC and suggests that inhibiting nuclear translocation of UBE2I may have therapeutic value in patients with ccRCC. Future research could explore the potential implications of UBE2I as a diagnostic and therapeutic target for ccRCC. For example, studies could investigate how blocking the nuclear translocation of UBE2I affects ccRCC cell viability, and explore the use of UBE2I as a diagnostic biomarker for ccRCC, potentially leading to better diagnosis and treatment options for patients with this disease. Overall, this article highlights the importance of identifying novel biomarkers for cancer diagnosis and treatment and demonstrates how CRISPR-Cas9 screening databases and immunohistochemistry can be used to identify potential targets for therapy.

In the next article, Sun et al. comprehensively discussed, through a literature review, the pivotal role of SUMO in topoisomerases, a class of enzymes that regulate the topology of DNA during essential processes including replication, transcription, recombination, and chromatin structure (Ju et al., 2022). This review sheds light on the intricate mechanisms by which topoisomerases manipulate DNA structures in double-stranded DNA to ensure genomic stability. Additionally, the authors introduce the critical role of SUMOylation in regulating the activity and interactions of topoisomerases. This review provides valuable insights into the molecular biology and genetics of DNA metabolism and is an excellent resource for researchers in the field. Of particular interest is the detailed discussion on the transesterification reaction between topoisomerases and DNA, which involves breaking of a phosphodiester bond in DNA by a topoisomerase enzyme, followed by the formation of a covalent bond between the enzyme and the broken end of the DNA strand. This process is essential for DNA strand relaxation and supercoiling and has implications for various cellular processes, including gene expression and DNA repair.

E-cadherin is a critical protein involved in cell adhesion that is frequently downregulated in cancer cells, resulting in increased invasiveness and metastasis. In the third article in this Research Topic, Sabatini et al. have demonstrated that the UBC9/SUMO pathway promotes the cleavage of E-cadherin, leading to increased invasiveness of HPV-positive head and neck cancer (Sabatini et al.). In this article, the clinical samples and biochemical assays demonstrated that SUMOylation of E-cadherin promotes the degradation of cleaved products. These findings shed light on the mechanisms underlying E-cadherin downregulation in HPV-positive head and neck cancer and provide potential targets for future therapeutic interventions. The authors identified the critical role of the UBC9/SUMO pathway in E-cadherin cleavage in HPV-positive head and neck cancer. Does the UBC9/SUMO pathway and its impact on E-cadherin cleavage have any effect on other HPV-positive cancers, such as anal and cervical cancers? Future studies could explore whether this pathway also plays an important role in other HPV-positive cancers. This research provides valuable insights into the molecular mechanisms driving cancer progression, emphasizing the importance of understanding these mechanisms to develop effective treatments for cancer patients.

In the next article, Li et al. conducted a study on the significance of SUMOylation genes in kidney cancer and their potential as biomarkers to predict prognosis and guide treatment selection (Li et al.). Researchers analyzed RNA expression data from kidney cancer tissues and developed a robust risk model, which was subsequently validated in multiple cohorts. The study identified differentially expressed SUMOylation genes in kidney cancer tissues, and constructed a risk model to predict patient outcomes and guide drug selection for personalized treatment based on RNA expression data. This approach has the potential to improve patient outcomes by identifying those at higher risk of a poor prognosis and selecting appropriate therapies based on their molecular profile. In conclusion, this study identified the importance of SUMOylation in cancer development and progression, and provided a promising avenue for personalized medicine in the treatment of kidney cancer.

Finally, in this Research Topic, Cao et al. provide a comprehensive overview of the role of SUMOylation in the regulation of RNA metabolism in cancer cells (Cao et al.). The authors summarize various ways in which SUMOylation affects RNA metabolism, including transcription, splicing, tailing, stability, and modification. They also highlight the impact of SUMOylation on microRNA biogenesis and function, which is particularly relevant to cancer development. The authors summarize that SUMOylation plays a critical role in regulating RNA metabolism and affecting various biological pathways, and understanding the mechanisms by which SUMOylation regulates RNA metabolism could lead to new therapeutic strategies for cancer treatment. Overall, this review provides a valuable resource for researchers in the intersection of RNA metabolism and cancer biology and offers insights into the complex interplay between the SUMOylation of proteins and cellular processes such as cell proliferation and apoptosis, which are key drivers of tumorigenesis and cancer progression.

Cancer is a global health concern that has garnered significant attention in the medical field (Siegel et al., 2023). Recently, remarkable advances in biotechnology-based precision cancer therapies have become mainstream in targeted cancer treatment (Yu and De Geest, 2020; Wahida et al., 2023). Specifically, immunotherapy combined with targeted small-molecule inhibitors has produced novel therapeutic regimens and renewed cancer research (Dagher et al., 2023; Vivekanandhan et al., 2023). The regulation of SUMOylation homeostasis of target proteins has emerged as a promising mechanism for targeted cancer therapy (Du et al., 2021).

In conclusion, it appears that abnormal levels of SUMOylation are closely associated with cancer development. Therefore, since the underlying mechanisms of SUMOylation in cancer occurrence and development continue to be elucidated, future research is expected to dive into greater detail and fundamental aspects in this area. It is anticipated that the development of small-molecule inhibitors targeting the homeostatic status of SUMOylation will facilitate the clinical treatment of cancer.
